# Locality, Realism, Ergodicity and Randomness in Bell’s Experiment

**DOI:** 10.3390/e25010160

**Published:** 2023-01-13

**Authors:** Alejandro Andrés Hnilo

**Affiliations:** CEILAP, Centro de Investigaciones en Láseres y Aplicaciones, (MINDEF-CONICET), J.B. de La Salle 4397, Villa Martelli 1603, Argentina; ahnilo@citedef.gob.ar

**Keywords:** quantum randomness, proposal of experiments on foundations of quantum mechanics, quantum key distribution security

## Abstract

Assuming that there is no way of sending signals propagating faster than light and that free will exists, the loophole-free observed violation of Bell’s inequalities demonstrates that at least one of three fundamental hypotheses involved in the derivation and observation of the inequalities is false: Locality, Realism, or Ergodicity. An experiment is proposed to obtain some evidence about which one is the false one. It is based on recording the time evolution of the rate of non-random series of outcomes that are generated in a specially designed Bell’s setup. The results of such experiment would be important not only to the foundations of Quantum Mechanics, but they would also have immediate practical impact on the efficient use of quantum-based random number generators and the security of Quantum Key Distribution using entangled states.

## 1. Introduction

It is well known that the violation of Bell’s inequalities is incompatible with the intuitive ideas of Locality and Realism. During the decades-long discussion on the experimental observations of that violation, it was argued that technical imperfections left space for the existence of some conspiratorial mechanisms, which received the general name of *loopholes*. These mechanisms were able to reproduce the observed results without contradicting Locality and Realism. However, loophole-free experiments [1,2,3,4,5,6] were performed and confirmed the violation of Bell’s inequalities. Therefore, at least one of the hypotheses necessary to derive and observe the violation must be false.

At this point, interpretations diverge. Some say that Locality and Realism mean essentially a single hypothesis, and what is false is “Local Realism” [7,8]. Others claim that only Realism is falsified, and that Locality plays no role in the problem [9]. In opposition, the expression *quantum non-locality* has become part of “popular knowledge”. Others argue that Quantum Mechanics (QM) is strictly Local [10,11,12], and that the violation of Bell’s inequalities is a consequence of the wavy nature of matter [13].

**Figure 1:**
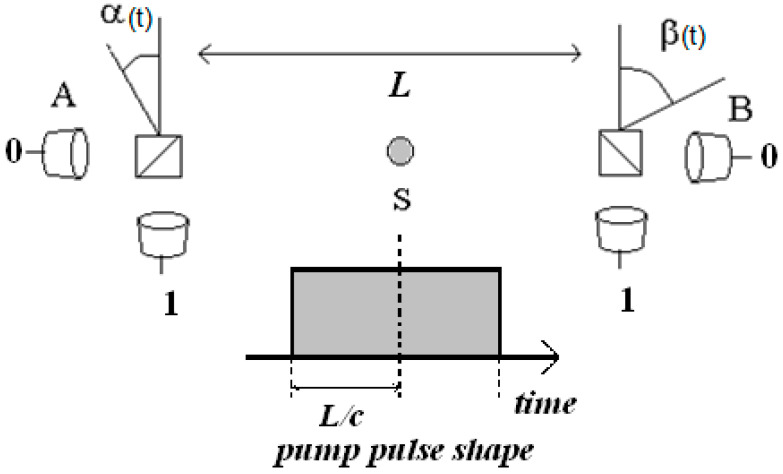
Sketch of a Bell’s setup and the proposed experiment. Source S emits pairs of photons maximally entangled in polarization, analyzers are set at angles {α,β} at stations A and B placed at distance *L*; single-photon detectors are placed at the output gates of the analyzers, producing binary time series. In the proposed experiment, pairs are emitted during well-separated pulses of duration ≈2*L/c*, settings {α,β} are changed just before the arrival of each pulse and remain fixed during the pulses’ duration. Hence, Local (i.e., spatially isolated) measurements in A and B are enforced during the first half of the pulses (t < *L/c*), but not during the second half (t > *L/c*). Measurements during the first (second) half of the pulses are hence performed in loophole-free (not loophole-free) condition. Therefore, observing the violation of Bell’s inequalities during the first half implies that at least one of the three hypotheses (Locality, Realism, and Ergodicity) necessary to derive and use Bell’s inequalities must be false. On the other hand, observing the violation during the second half is possible even if the three hypotheses are true. Depending on which one of the three hypotheses is false, the level of randomness of the series recorded in the first and second halves may be different.

There is an additional twist: Locality and Realism suffice to derive Bell’s inequalities, but, in order to test them experimentally, an additional hypothesis is necessary. This was originally shown by V. Buonomano in 1978, who named it *Ergodicity* [14]. The necessity of the additional hypothesis was rediscovered during the years with different names: homogeneous dynamics, uniform complexity, experiments’ exchangeability, counter-factual stability [15,16,17,18], and there are probably more that escaped my attention. The many versions of this hypothesis have subtle differences, but, at the end of the day, they all mean the following: that it is possible to insert data measured with different angle settings (see Figure 1), which are unavoidably recorded at different values of time, into a single theoretically derived expression (i.e., the Bell’s inequality). Details on the necessity and meaning of this hypothesis are discussed in Section 2. I stress that it is not necessary to derive Bell’s inequalities, but that it is unavoidable to insert measured data into them.

In order to avoid confusion, the meanings of “Locality” and “Realism” as they are understood *in this paper* are also defined in Section 2. I do not claim they are the “correct” or “best” definitions. They are just the ones used in this paper. According to these definitions, Locality, Realism, and Ergodicity are separate hypotheses, all of them necessary to derive and observe Bell’s inequalities.

Strictly speaking, two other hypotheses are also necessary: *freedom of choice* (which is relevant here in choosing the angle settings in Figure 1 by the observer) and *non-signaling* (i.e., the impossibility of sending signals that propagate faster than light). I assume these two hypotheses are valid. I also assume that all loopholes are closed.

In these conditions, a relevant question is: which one of the three remaining fundamental hypotheses involved (Locality, Realism, and Ergodicity) is false when Bell’s inequalities are experimentally violated? Of course, more than one can be false. The cases where only one of them is false are considered here. Therefore, in the situation the falsity of (f.ex.) “Locality” is considered, it is implicitly assumed that “Realism” and “Ergodicity” are true. The aim of this paper is to propose an experiment to reveal (or, at least, to obtain some evidence to indicate) the false hypothesis. The key is the relationship between *falsity* of each one of the three hypotheses and *randomness* of the series of outcomes produced in a Bell’s setup. The problem of the definition and testing of randomness is reviewed at the end of Section 2. In Section 3, it is reviewed that the falsity of Locality implies the series must be “truly” random, and that the falsity of Realism leaves the series’ randomness undecided. Also in that Section, I claim that the falsity of Ergodicity implies the series must be non-random. In consequence, an experiment testing the series’ randomness is, in principle, able to reveal the false feature. However, several problems must be considered. An attainable experiment is described in Section 4.

To avoid any confusion, the proposed experiment does not affect the validity of QM. It is not the proposal of a new test of QM. What is proposed to test here is which hypothesis among Locality, Realism, or Ergodicity (or some hypothesis with the same consequences as Ergodicity) is false. The results of the proposed experiment may affect the interpretation of QM (f.ex., the Copenhagen interpretation assumes that Realism is false), but not the validity of QM or its predictions.

## 2. Definitions Used in This Paper

### 2.1. Realism

There are many definitions of Realism. The issue is subtle and complex [19]. In this paper, Realism means that it is possible to write the probability of observing a given outcome in Figure 1 as an average over classical probabilities and distributions on a hidden (but classical, counterfactually definite) variable λ. F.ex, the probability of observing the outcome “1” in station A when the setting is α, is assumed to be

P_A_^(1)^(α)*_assumed_* = ∫dλ.ρ(λ).P_A_^(1)^(α,λ) (1)

where both the distribution ρ(λ) and the probability P_A_^(1)^(α,λ) hold to the axioms of classical (Kolmogorov’s) theory of probability, and the integral is a Lebesgue or Riemann one. Expressions such as Equation (1) are at the basis of the derivation of Bell’s inequalities.

### 2.2. Locality

It is worth mentioning here that “Locality” is often defined as the assumption that the probabilities of coincidences (at the hidden variables level) are statistically independent, f.ex.:

P_AB_^(1,0)^(α,β,λ) = P_A_^(1)^(α,λ) × P_B_^(0)^(β,λ) (2)

Equation (2) uses classical probabilities, so that this definition of Locality presupposes the definition of Realism above. That is why the term “Local Realism” is appropriate if Equation (2) is chosen to define Locality.

In this paper, instead, “Locality” is defined simply as the non-existence of effects propagating faster than light. If a perturbation is introduced by Bob in the system at B, that perturbation cannot be the cause of changes (neither observable nor hidden) in the system at A before a time longer than *L/c* has elapsed (the stations are in the same system of reference, so that “time” is well defined). Nothing is said about hidden variables or probabilities. This definition of Locality is hence independent of Realism as it is defined in Section 2.1. Yet, if Realism is assumed valid, then this definition of Locality implies that Equation (2) is valid.

### 2.3. Ergodicity

There are several ways to see the necessity of a hypothesis additional to Locality and Realism in order to use Bell’s inequalities in experiments [14,15,16,17,18]. F.ex., in the derivation of the Clauser–Horne (CH) inequality, an algebraic relationship leads to the following inequality (the super index is dropped here, for the CH inequality involves one detector per station only):

−1 ≤ P_A_(α,λ).P_B_(β,λ) − P_A_(α,λ).P_B_(β’,λ) + P_A_(α’,λ).P_B_(β,λ) + P_A_(α’,λ).P_B_(β’,λ) − P_B_(β,λ) − P_A_(α’,λ) ≤ 0 (3)

This equation is then multiplied by the classical distribution ρ(λ) and integrated over the space of the hidden variable as in Equation (1) to obtain the final expression of the CH inequality:

−1 ≤ P_AB_(α,β) − P_AB_(α,β’) + P_AB_(α’,β) + P_AB_(α’,β’) − P_B_(β) − P_A_(α’) ≡ J ≤ 0 (4)

However, all real measurements occur during time. The expression of the observable probabilities are (f.ex., for P_A_ in Equation (1))

(5) $$P_{A}(\alpha )=(1/\Delta t)\int_{\theta }^{\theta +\Delta t}dt.\rho (t).P_{A}(\alpha ,t)$$

This equation represents the result of the following real process: set A = α during the time interval [*θ*,*θ +* Δ*t*], sum up the number of photons detected after the analyzer, and obtain P_A_(α) as the ratio of detected over incident photons. Note that Equation (5) is equivalent to Equation (1) if “time” is interpreted as the hidden variable. Following the usual reasoning leading to the CH inequality, time is now integrated to obtain

(6) $$-T\leq \int_{0}^{T}dt.\rho (t).P_{AB}(\alpha ,\beta ,t)-\int_{0}^{T}dt.\rho (t).P_{AB}(\alpha ,\beta^{\prime },t)+\int_{0}^{T}dt.\rho (t).P_{AB}(\alpha^{\prime },\beta ,t)+\int_{0}^{T}dt.\rho (t).P_{AB}(\alpha^{\prime },\beta^{\prime },t)\;-\int_{0}^{T}dt.\rho (t).P_{B}(\beta ,t)-\int_{0}^{T}dt.\rho (t).P_{A}(\alpha^{\prime },t)\leq 0$$

where *T* is the total time of observation. This inequality is certainly valid, but it does not correspond to what is actually measured, as it is impossible measuring with two different settings (say, α and α’) at the same time. In most experiments on Bell’s inequalities, the measuring time is distributed in a way similar to the following: the analyzer A is set to α between *t* = 0 and *t* = *T*/2 and to α’ between *t* = *T*/2 and *t* = *T*, B = β between *t* = *T*/4 and *t* = 3*T*/4, and B = β’ between *t* = 0 and *t* = *T*/4, and also between *t* = 3*T*/4 and *t* = *T*. A different distribution (f.ex.: a random fast variation of the settings) requires a more involved notation of the integration intervals, but the result is the same. The value of J (see Equation (4)) that is actually measured in an experiment is then:

(7) $$(1/\Delta T)\int_{T/4}^{T/2}dt_{2}.\rho (t_{2}).P_{AB}(\alpha ,\beta ,t_{2})-(1/\Delta T)\int_{0}^{T/4}dt_{1}.\rho (t_{1}).P_{AB}(\alpha ,\beta^{\prime },t_{1})+(1/\Delta T)\int_{T/2}^{3T/4}dt_{3}.\rho (t_{3}).P_{AB}(\alpha^{\prime },\beta ,t_{3})\;+(1/\Delta T)\int_{3T/4}^{T}dt_{4}.\rho (t_{4}).P_{AB}(\alpha^{\prime },\beta^{\prime },t_{4})-(1/2\Delta T)\int_{T/4}^{3T/4}dt^{\prime \prime }.\rho (t^{\prime \prime }).P_{B}(\beta ,t^{\prime \prime })-(1/2\Delta T)\int_{T/2}^{T}dt^{\prime }.\rho (t^{\prime }).P_{A}(\alpha^{\prime },t^{\prime })$$

(Δ*T =* T*/4*) which is different from Equation (6), breaking the logical chain in the derivation of the inequality. In order to retrieve the validity of the usual CH inequality in the experiments, it is necessary to assign (hypothesize) numerical values to counterfactual results, that is, the values of the integrals that would have been observed, during a certain time interval, if the settings had been different from the ones that were actually used [15,20]. The hypothesis that is *tacitly* made in the experiments is that the values of measured averages (f.ex.):

(8) $$P_{A}{\left(\alpha \right)}_{\mathrm{measured}}=(1/T).\int_{\theta_{i}}^{\theta_{i}^{}+T}dt.\rho (t).P_{A}(\alpha ,t)$$

are independent of *θ_i_* (for sufficiently long *T*). In other words: that the value obtained with *θ_i_*, when detections were recorded with actual setting α, is equal to the one that would have been obtained with *θ*’*_i_*, when the actual setting was α’. This tacit hypothesis (or an equivalent one, as many authors independently found) is unavoidable when inserting measured data into the derived expressions of Bell’s inequalities.

Buonomano realized that the tacit hypothesis could be justified in a physically sound way by assuming Ergodicity. The argument can be briefed as follows: Note that Equation (1) is an integral over the space of states of the hidden variable. It is an *ensemble* average. Equation (8), instead, is a *time* average. Ergodicity valid means that ensemble and time averages are equal, i.e., that Equations (1) and (8) are equal. As there exist only one P_A_(α)*_assumed_*, then all P_A_(α)*_measured_* have the same value, regardless of the values of *θ_i_* that are recorded. Therefore, assuming Ergodicity valid implies, in physically meaningful terms, the validity of the tacit assumption, and allows the use of Bell’s inequalities in experiments. Be aware that Ergodicity has no relationship with the memory or any other loophole (supposing the opposite was an error in Buonomano’s paper). In this paper, all loopholes are assumed closed.

### 2.4. About Randomness

There is no unanimously accepted definition of randomness. It is only agreed that “predictable” (even partially predictable) implies “not random”. Nevertheless, levels of randomness have been established [21]:

*Uniformity, or Borel-normality* means that the statistical average rate of strings of length *n* (say, 110100 for *n* = 6) in the series is the same as would be obtained by tossing an ideal coin. Uniformity is a necessary, but not sufficient, condition for randomness [22]. Yet, certifying this property is difficult. Other statistical tests of randomness involve the decay of the self-correlation or the mutual information. They all involve measuring probabilities, and hence require the series to be stationary. The battery of tests provided by the National Institute of Standards and Technology (NIST) is mostly based on this approach.

A series can pass the statistical tests just mentioned and still be predictable, then, not random. A well-known example is the series of the binary digits of π, which is generated by an algorithm. A series is *algorithmically random* if there is no algorithm able to generate the series using a number of bits shorter than the said series. Note that this definition does not involve probabilities. It applies even to series that are not statistically stationary. Algorithmic randomness is related to Kolmogorov’s complexity [23]. The complexity *K* of a binary series of length *N* is the length of the shortest program, running on a classical Turing machine, whose output is the said series. Therefore, a series is algorithmically random if *K* ≈ *N*. This definition is intuitive, free of ambiguities, and appealing, but has a serious drawback: *K* cannot be actually computed, for one can never be sure that there is no shorter program able to generate the series. It can be only *estimated* from the compressibility of the series by using, f.ex., the algorithm devised by Lempel and Ziv [24].

On the other hand, Martin-Löf’s theorem ensures that there exists a *universal algorithmic test* that determines if a given series is random, at least in the typical and algorithmic senses [25]. Unfortunately, the expression of this universal test is unknown. An approach at hand is as follows: a given series can be demonstrated *non*-random. This occurs when it is rejected by one of the many existing tests of randomness, both statistical (f.ex., the NIST battery of tests) and algorithmic (f.ex., estimators of Kolmogorov’s complexity). As the number of applied tests is increased, the result that would be obtained by applying the unknown universal test is approached, say, asymptotically. This is known as *Ville’s principle*, and is used to evaluate the reliability of random number generator (RNG) codes or devices in practice. A high rate of rejected series, or *rejection rate*, means a low level of randomness. The rejection rate does not properly measure randomness, as the set of applied tests is arbitrary. Yet, it is evident that it cannot be *completely unrelated* from “actual” randomness, as defined by the (unknown) universal algorithmic test. As it will be shown, a coarse relationship between the rejection rate and “actual” randomness is all that is needed for the aims of this paper.

## 3. Consequences of Non-Validity of Each Hypothesis

### 3.1. Non-Locality and Randomness

According to the idea of Quantum Certified Randomness (QCR), the binary series of outcomes produced in the Figure 1 setup are *intrinsically* random. As “random” is a difficult feature to define, the idea of QCR is most appealing. The setup in Figure 1 would provide then not only series to be used in practice, but also a definition: *random series is what is produced by this setup*. To my knowledge, QCR is supported by three different arguments:

(i) Because of a numerical relationship, the parameter S_CHSH_ (which is a usual measure of entanglement) puts a lower bound to the series’ minimum entropy H*_min_* [26]. If S_CHSH_ reaches its maximum value 2√2, then H*_min_*= 1, which is the minimum entropy of an ideally uniform series.

(ii) Arguments of the Kochen–Specker type show that the outcomes of some quantum experiments cannot be assigned by a program running on a classical Turing machine [27,28]. That is, they are *Turing non-computable*.

(iii) If the existence of non-Local effects is taken *as an axiom* (i.e., if Locality as defined in Section 2.2 is false) then the series of outcomes produced by measurements on a spatially spread entangled state cannot be predicted by any method. Otherwise, faster than light signaling would be possible [29].

The argument (i) above guarantees a minimum level of uniformity of the series, but, as said, a series can be ideally uniform (H*_min_* = 1) and still be predictable and hence, not random. Regarding (ii), a series can be Turing non-computable and still not algorithmically random. Incomputability is a necessary, but not sufficient “symptom” of “true randomness” [28]. The argument (iii) is the strongest; it ensures that no algorithm can predict the series. I will assign “random” to this strongest condition only.

In short, if Bell’s inequalities are violated because Locality (as defined in Section 2.2) is false, then, according to the arguments in [29], the series produced in Figure 1 must be (algorithmically) random.

### 3.2. Non-Ergodicity and Randomness

Let us hypothesize that the series of outcomes in Figure 1 are caused by the evolution of an underlying classical dynamical system. I find intuitive the relationship between Ergodicity and randomness in this context. A classical system that evolves ergodically fills the phase space evenly. If the evolution is non-Ergodic instead, then the system spends more time in some regions of phase space than in others of the same measure (because, time and ensemble averages are supposed not equal). Hence, at a given time, the system is more probably found in some regions than in others. Its future state can be partially predicted. Therefore, an evolution that is non-Ergodic is (at least partially) predictable and hence non-random, for all definitions of “random”.

In more formal terms, attempts to explain the violation of Bell’s inequalities within classical Physics assume that the evolution of an underlying classical dynamical system causes the outcomes in Figure 1. Let us partition the phase space of this system as follows: Label “1”(“0”) the regions where the system causes a “1”(“0”) in the series. Inside these regions, there are sub-regions where the system causes the strings 11, 10 (01, 00). Following this partition up to some arbitrary large number *n* (*n* is nevertheless much shorter than the total length of the series), the phase space is divided into 2*^n^* sub-regions. Actual series are finite, so that there is always a finite value of *n*. When the system evolves into one of these sub-regions, the corresponding string appears in the series.

Birkhoff’s theorem ensures that Ergodicity is valid *if and only if* the phase space is metrically un-decomposable. Therefore, if Ergodicity is not valid, then the phase space is metrically decomposable. This means that it can be divided into two regions of measure different from 0 or 1 that are invariant during the system’s evolution [30]. The system’s evolution is then trapped into one of these regions, and it never enters into the other one. The invariant regions have measures different from 0 or 1, hence they include a finite number of the 2*^n^* labeled sub-regions, which are never visited by the system. In consequence, there are a finite number of strings of length *n* that do not appear in the complete series. The complete series is then, by definition, not uniform. As uniformity is a necessary condition for randomness, a non-Ergodic evolution (of the assumed underlying classical dynamical system) causes non-random series.

Be aware that this reasoning applies only to the case of interest here, that is, a classical system with a bounded phase space which produces a binary series as it enters different regions of its phase space. In this case, and in this case only, if the evolution of the system is not Ergodic, then the produced series is not uniform (and hence, not random). The relationship not Ergodic ⇒ not random is not claimed to be general. For example, the (unbounded) random walk is random (by definition) and non-Ergodic.

### 3.3. Non-Realism and Randomness

The Copenhagen interpretation of QM is the most important of the descriptions of the violation of Bell’s inequalities that hypothesizes Realism to be false. Contrarily to usual belief, this interpretation says *nothing* about the series’ randomness. Born’s rule allows calculating probabilities (defined as the limit of frequencies), but is silent about the features of the series that underlie the measurement of such probabilities. The only explicit opinion on this subject is von Neumann’s axiom. It states that quantum measurements violate Leibniz’s principle of sufficient reason: the outcome “1” or “0” in Figure 1 have no cause. A series of such outcomes is intuitively random, but this intuition is difficult to formalize [22]. In addition, von Neumann’s axiom can be understood in two ways, or strengths. Its “strong” form means that Leibniz’s principle is violated in quantum experiments. The “weak” form means that the axiom is part of a user’s guide or warning about what QM can or cannot predict, but not necessarily a feature to be experimentally observed. In addition, algorithmic randomness of quantum produced series has not been established [28].

In short, if Bell’s inequalities are violated because Realism is false, then, according to the arguments in [22,28], there is no reason to say that the produced series are (algorithmically) random, or not.

## 4. Proposed Experiment

### 4.1. Basic Scheme

As discussed in the previous sections, the falsity of each of the three main hypotheses (as they are defined in Section 2) implies different randomness of the series produced in the setup of Figure 1. Locality false implies the series must be algorithmically random, Ergodicity false implies that they must be non uniform (hence, not random), and Realism false leaves the series’ randomness undecided. This result opens a way to decide which hypothesis is false. However, nothing can be assumed about the validity of any of the three involved hypotheses (otherwise one would fall into a logical inconsistency) so that the rate of rejected series (Ville’s principle) appears as the available method to evaluate randomness.

In practice, the rejection rate can be affected by many “technical” causes. The challenge is to find an experimental approach that gets rid of these causes, leaving only the effect of the falsity of one of the hypotheses. As always, the *relative variation* of a magnitude (in this case, randomness) is much easier to measure than its absolute value.

Suppose then that the source in Figure 1 emits maximally entangled states during square pulses of total duration twice longer than *L/c*, where *L* is the distance between stations and *c* is the speed of light. Time between pulses is adjusted to be much longer than the pulses’ duration. Intensity is adjusted such that much less than one photon per pulse is recorded in average. Trigger signals are sent to each station to indicate the start of each pulse and synchronize the clocks. Angle settings {α,β} in each station are “randomly” (see Section 4.5) changed (as in the loophole-free experiments) just before the arrival of each pulse, and then they are left fixed during each pulse. Time-to-digital converters record the time elapsed from the start of each pulse until the detection of each photon. This is repeated for many (typically, tens of millions) pulses during an experimental run. After the run has ended, data processing identifies the coincidences between A and B. Single detections are discarded. Binary series for each time interval within the (say, stroboscopically reconstructed) pulse are obtained in this way. The size of the time intervals depends in practice on the number of recorded coincidences (see the end of Section 4.3). In the discussion that follows, only two time intervals are considered: the pulses’ first half and the pulses’ second half.

Suppose now that the violation of Bell’s inequalities is constant during the pulse duration, as predicted by QM and also observed [31,32]. During the pulses’ first half, detections at A and B are spatially isolated. Therefore, during the pulses’ first half, the violation of Bell’s inequalities is possible only because Locality, *or* Realism, *or* Ergodicity is false. We *know* that one of them must be false. During the pulses’ second half instead, there has been enough time for classical information to propagate between the stations, and Bell’s inequalities can be violated even if the three hypotheses are true. This experimental feature implies that the level of randomness during the first half may vary with respect to the one in the second half, and that this occurs in a time typically too short for other perturbations (mechanical or thermal) to affect the results. The type of variation to be expected depends on which hypothesis is false, as it is discussed next.

### 4.2. If Locality Is False

Let us suppose that the violation of Bell’s inequalities observed during the pulses’ first half occurs because Locality is false. Therefore, as reviewed in Section 3.1, series recorded during the first half *must* be algorithmically random. Instead, series recorded during the pulses’ second half may be random, or maybe not. It is natural to expect the level of randomness, averaged over a large set of data, to decrease when passing from an enforced random regime to a non-enforced one. Therefore, the rejection rate averaged over large statistical samples should *increase* from a value near to zero for the sample recorded during the pulses’ first half (loophole-free enforced), to a non-negligible value for the sample recorded during the pulses’ second half (loophole-free not enforced). Note that only a coarse relationship between the rejection rate and “actual” randomness (as defined by the unknown universal algorithmic test) is assumed, that is, that they either increase or decrease together (not necessarily in the same amount) in the average.

### 4.3. If Ergodicity or Realism Are False

Let us suppose now that the violation of Bell’s inequalities observed during the pulses’ first half occurs because Ergodicity is false. Series recorded during the first half *must* be non-uniform now. The rejection rate should be close to 100%. Instead, series recorded during the second half may be uniform, or maybe not. Following the same reasoning than in the previous Section, the rejection rate in the sample of series recorded during the pulses’ second half should now *decrease*.

Finally, let us suppose that the violation of Bell’s inequalities observed during the pulses’ first half occurs because Realism is false. Series recorded in the pulses’ first half may be random or maybe not. The same applies to the series recorded in the second half. Therefore, the rejection rate averaged over large samples should remain constant during the pulse duration.

Usual sources of non-randomness, like different detectors’ efficiencies, are of course constant during the pulse duration. If the pulses are short enough (see Section 4.5), any thermal or mechanical perturbation will affect the rejection rate in the same way during the whole pulse duration. The variation of the rejection rate between the first and second halves, caused exclusively by the falsity of one of the hypotheses, should then be detectable in a statistically meaningful sample of series.

In summary, the consistent observation of an increasing (decreasing) rejection rate during the pulse duration suggests Locality (Ergodicity) is false. A constant rate suggests Realism is false instead. Conceivably, the latter result can also be caused in practice by a high level of noise masking the actual trend. In the case that the trend is in fact observed to be constant within statistical deviation, the influence of sources of noise existing in the actual setup should be carefully analyzed. In order to help estimate the statistical deviation, the rejection rate should be calculated for different choices of the sets of tests (see next Section).

If sufficient data are available, the pulses can be sliced in more than two parts (i.e., more than two time intervals, see the end of Section 4.1) and a *curve* of evolution of the rejection rate during the pulse duration can be plotted. This would allow the study of statistical correlation in a complete way and the reaching of more reliable conclusions.

### 4.4. Tests of Randomness and a Practical Consequence

A usual choice to apply Ville’s principle is the NIST battery of 16 statistical tests. As said, it is convenient using a set of tests as large and diverse as possible. It is possible to add estimators of Kolmogorov’s complexity [33] and tools of nonlinear analysis to identify a compact object in phase space (Takens’ theorem) [34]. Entropies can be calculated. This is just an example of the set of tests that can be used.

For the aims of the proposed study, evaluating randomness according to the Ville’ principle has the crucial advantage that no assumption about the validity of Locality, Realism, or Ergodicity is made. On the other hand, the measured rejection rate depends on the set of tests chosen, which is arbitrary. For this reason, I claim the consistent observation of a trend in the time variation of the rejection rate to provide *some evidence* about the falsity of one of the hypotheses, not a *proof*.

In spite of this limitation, the result of the proposed experiment has an immediate practical impact. Pulsed sources are useful in QKD to reduce signal-to-noise ratio and to synchronize the clocks, which is a technical problem of main concern. If the rejection rate was shown to increase with time, then QKD using entangled states would be safer if pulses shorter than *L/c* were used to generate the key. If the rejection rate was shown to decrease instead, the final part of long pulses (duration > *L/c*) should be preferred. Finally, if the rejection rate was shown to be constant, then both the pulse duration and the pulse’s part used would be irrelevant. Similar advice would apply to the most efficient way (i.e., with the lowest number of non-random series delivered) to operate a pulsed quantum RNG. Note that this practical advice would be valid even if the foundational issue remained not fully decided.

### 4.5. Conditions for an Attainable Experiment

Unfortunately, the experiment as described is unattainable nowadays. Due to detectors’ efficiency, the loophole-free violation of Bell’s inequalities can be reached with photons only by using Eberhardt’s states, which produce non-uniform series. Extractors of randomness are applied [35,36], but their use in this case may mask the trend that it is intended to reveal. Setups exploiting entanglement swapping between photons and matter do use Bell states, but produce a rate of detections too low to be suitable.

A simple solution at hand is to accept the *fair sampling* assumption [37] as valid. This means that the set of recorded coincidences is an unbiased statistical sample of the whole set of detected and non-detected photons. Under this assumption, Bell states and existing photon detectors can be used.

Other problems are achieving fast and random setting changes between the pulses. In addition to the technical difficulty of fastness, there is the logical problem (a sort of infinite regress) of performing *random* setting changes. Both problems can be circumvented by assuming that any hypothetical correlation between A and B vanishes when the source of entangled states is turned off. This assumption is supported by the following observation: in a pulsed Bell’s setup, the S_CHSH_ parameter is observed to decay following a certain curve if the time coincidence window is increased beyond the pulse duration. This curve fits the one that is predicted if the detections outside the pulse are assumed to be fully uncorrelated [31]. Assuming non-correlation implies the curve but, of course, observing the curve does not necessarily imply non-correlation. Nevertheless, if the latter implication (which is most reasonable) is accepted as true, then random settings’ changes become unnecessary. Only pulses well separated in time are required.

*Under these two assumptions* (“fair sampling” and, say, “uncorrelated when the source is turned off”) the proposed experiment is at hand even with limited means. The results obtained in these conditions may not be considered definitive, but they may still give a clue about the answer to the main question. They may also help to decide whether or not it is worth the effort to make the complete experiment. Also important, they would have an immediate practical impact (see the end of Section 4.4 above).

In order to keep the rate of accidental coincidences low, source intensity or pumping power must be adjusted such that the probability *p* of detection per pulse is *p* << 1 [38]. The photon down-conversion process is usually so weak, and collection efficiency of radiation so limited, that this condition is easily reached in practice for the pulse duration of interest here. In other words, most pulses are “naturally empty”. Choosing *p* = 0.1 and pulse repetition rate 1 MHz, series 6 Mbits long are then recorded at each station in a run lasting 300 s. It is not convenient to increase the repetition rate beyond that value, as ≈1 MHz is the typical threshold of saturation of available single-photon detectors (silicon avalanche photodiodes). If the stations are separated by 20 m, then the pulse duration is ≈120 ns and duty cycle is ≈12%. These numbers are easily achievable by pumping the nonlinear crystals that generate the entangled states with a pulsed diode laser, which typically has a bandwidth of 20 MHz. Samples of the laser pulses can be sent to each station and recorded with fast photodiodes to synchronize the clocks of the recording devices. Detectors’ blind time has been identified to cause non-uniform series in some quantum RNG. But this is not important in the proposed experiment, because the average number of detections per pulse is, as said, adjusted to be small (*p* << 1) and pulses are well separated.

## 5. Summary

By recording the coarse time evolution of the rate of non-random series obtained in a suitable pulsed Bell’s experiment, it is possible to obtain some evidence about which one among three fundamental hypotheses (Locality, Realism, or Ergodicity) is false when the violation of Bell’s inequalities is observed. This is of obvious interest for the foundations of QM.

The proposed experiment requires some additional assumptions to be technically achievable nowadays. This may weaken its impact from the foundational point of view. Nevertheless, measuring the variation in the rate of non-random series within the pulses would have immediate practical impact on the best use of quantum RNG and of device-independent QKD. Depending on the experiment’s result, it may be advisable to use sources with pulses shorter than *L/c*, or instead use the end of long pulses (>*L/c*), or it may also turn out that the pulses’ duration and selected section are irrelevant.

## Data Availability

No research data are available.
